# Contribution of Polymorphisms in IKZF1 Gene to Childhood Acute Leukemia: A Meta-Analysis of 33 Case-Control Studies

**DOI:** 10.1371/journal.pone.0113748

**Published:** 2014-11-25

**Authors:** Yue-e Dai, Linjun Tang, Jasmine Healy, Daniel Sinnett

**Affiliations:** 1 Nanjing Children's Hospital, Nanjing Medical University, Nanjing, China; 2 Department of Neurosurgery, Tongling People's Hospital, Tongling, Anhui, China; 3 Sainte-Justine University Hospital Research Center, Montreal, Quebec, Canada; 4 Department of Pediatrics, Faculty of Medicine, University of Montreal, Montreal, Quebec, Canada; University of Texas, United States of America

## Abstract

**Objective:**

Two common polymorphisms in the IKZF1 gene (rs4132601 and rs11978267 variants) have been reported to be associated with childhood acute leukemia (AL) risk, however the results were inconsistent. Here, we conducted a meta-analysis to generate large-scale evidence on whether IKZF1 variants are risk factors for childhood AL.

**Methods:**

The PubMed, Embase, EBSCO, and Web of Science were searched up to June 2, 2014 for studies on the association of IKZF1 polymorphisms with childhood AL risk. Data were extracted and the odd ratios (ORs) and95% confidence intervals (95% CIs) were calculated by a fixed-effects orrandom-effects model. Subgroup analysis by ethnicity and leukemia subtype, sensitivity and cumulative meta-analyses were performed. Moreover, publication bias was assessed by Begg's and Egger's tests.

**Results:**

In total, 33 case control studies were finally included in this meta-analysis. For rs4132601 polymorphism, significantly increased AL risk was observed in all genetic models (the association was still significant when the p value was Bonferroni adjusted to 0.025). In the subgroup analysis by tumor type, statistical association was observed in B-cell precursor ALL (BCP-ALL). Additionally, when stratified by ethnicity, significantly increased AL risk was only observed in European subgroup, but not among African or mixed population subgroups. Finally, similar results were found forrs11978267 polymorphism.

**Conclusion:**

In summary, this meta-analysis provides evidence that rs4132601 and rs11978267 polymorphisms in the IKZF1 gene mightcontribute to the occurrence of BCP-ALL, especially in European populations. Moreover, further studies with large sample size are required to clarify possibleroles of IKZF1 variants in other ethnic groups (e.g., Asians and Africans).

## Introduction

Acute leukemia, the most common type of childhood cancer and the leading cause of cancer-related deaths among children, affects 35–50 per 1,000,000 children per year [1], Acute leukemia is usually subdivided into two clinical forms according to cell morphology, immunophenotype and cytogeneticas characteristics in acute lymphoid leukemia (ALL) and in acute myeloide leukemia (AML) [2]. The peak onset of acute leukemia occurs at 2 to 5 years of age [3]. Previous studies showed that initiation of leukemogenesis occurs during fetal life or in early infancy and is likely caused by multiple factors [4], nevertheless, the exact mechanisms underlying the development of this hemotologic malignancy remains poorly understood.

Recently, accumulating studies suggest that inherited genetic factors affect the risk of developing ALL. Two genome-wide association (GWA) studies have identified SNPs in 7p12.2 (IKZF1), 9p21 (CDKN2A), 10q21.2 (ARID5B), and 14q11.2 (CEBPE)that contribute to susceptibility to ALL [5], [6]. IKZF1encodes the early lymphoid transcription factorIKAROS, which is a DNA-binding zinc finger transcription factor involved in the development of all lymphoid lineages. However, several following replication studies could not validatethe association between polymorphisms (rs4132601 and rs11978267) in IKZF1 gene and acute leukemia risk [7]–[11]. This contradiction might be attributed to, at least in part, small sample sizes and ethnic differencesaccross studies.

To date, one meta-analysis focused on the correlation between IKZF1 variants and ALL risk, which only investigated the association of one polymorphism (rs4132601) and ALL risk in the overall population. Moreover, some studies involving childhood acute leukemia were not included [8], [10]–[13]. Thus, we performed a meta-analysis, which provided more credible evidence by systematically summarizing all eligible data, to clarify the effects of two IKZF1 polymorphisms (rs4132601 and rs11978267) on childhood ALL as well as AML risk.

## Materials and Methods

### Study identification and eligibility criteria

We systematically searched PubMed, Embase, EBSCO, and Web of Scienceusing the following search terms:(‘acute leukemia’, ‘acute myeloid leukemia’, ‘acute myeloblastic leukemia’, ‘AML’, ‘ALL’ or ‘acute lymphoblastic leukemia’),(‘IKZF1’, ‘rs4132601’ or ‘rs11978267’) and (‘polymorphism’, ‘variant’, ‘mutation’). The search was last performed in June 2014. Moreover, the reference lists of retrieved articles were checked for additional potential studies.

A study was eligible in the meta-analysis if it: (1) investigated the association of IKZF1 polymorphisms with childhood acute leukemia susceptibility (2) provided sufficient data on allele or genotype distribution in patients and controls. The exclusion criteria were: (1) no control population (2) the subjects of the study were adults (3) comments, review articles, meta-analysis, or articles only with an abstract.

### Data extraction

From each study, the following data was extracted independently by two authors: first author, publication year, country and ethnicity of the subjects, gender component, mean age of the study subjects, genotyping method, number of patients and controls, types of acute leukemia, allele and genotype frequency of patients and controls. In addition, if the genotype distribution was unavailable in the article, the corresponding author was contacted for the detailed data. Disagreements were resolved by discussion between the two investigators.

### Quality score assessment

The quality of each study was independently assessed by 2 authors using the quality scoring scale modified from previous meta-analysis of genetic studies [14]–[16]. These quality score of a given study were based on both traditional epidemiologic considerations and genetic issues. (Table S1) Total quality scores ranged from 0 points (worst) to 12 points (best), and a study was considered high quality if score was 8 points or higher.

### Statistical analysis

The strength of the association between IKZF1 polymorphisms (rs4132601 or rs11978267) and childhood acute leukemia was measured by odds ratios (ORs) and corresponding 95% confidence intervals (CIs). The significance of the pooled OR was determined by the Z-test, and the P values were adjusted using Bonferroni correction by the number of compared SNPs (p = 0.05/2 = 0.025).Stratified analysis was performed according to types of AL (B-cell precursor ALL (BCP-ALL), T-cell ALL and AML) and ethnicity (Europeans, Asians, Africans). Additionally, the Hardy-Weinberg equilibrium (HWE) of the control group was assessed, and a P value of less than 0.05 was considered significant disequilibrium.

Heterogeneity across studies was assessed by χ^2^-based Q test and I^2^ test, and heterogeneity was considered significant when a P value was less than 0.10 [17], [18]. A fixed effects model was used when the heterogeneity was non-significant; otherwise, a random effects model was used [19]. Galbraith plot, which identifies the outliers as possible sources of heterogeneity, was used to visualize the impact of individual studies on the overall homogeneity [20]. Moreover, meta-regression was also performed to explore the possible heterogeneity among different kinds of studies. The parameter τ^2^ in meta-regression is the residual between-study variance that describes the variation in the results that is not explained by the covariates [21], [22].

Sensitivity analysis was performed by sequentially omitting one study each time to assess the effect of a single study on the pooled ORs. In addition, cumulative meta-analyses were also carried out for both variants in association with AL to evaluate the trend of the genetic risk effect (OR) of the allele contrast as evidence accumulating over time. Finally, publication bias was assessed using graphical evaluation of Begg's funnel plots and the Egger's regression test, a p value of less than 0.05 was considered as significant [23], [24]. All statistical analyses were performed by STATA software, version 12 (StataCorp LP, College Station, Texas).

## Results

### Characteristics of eligible studies

**Figure 1:**
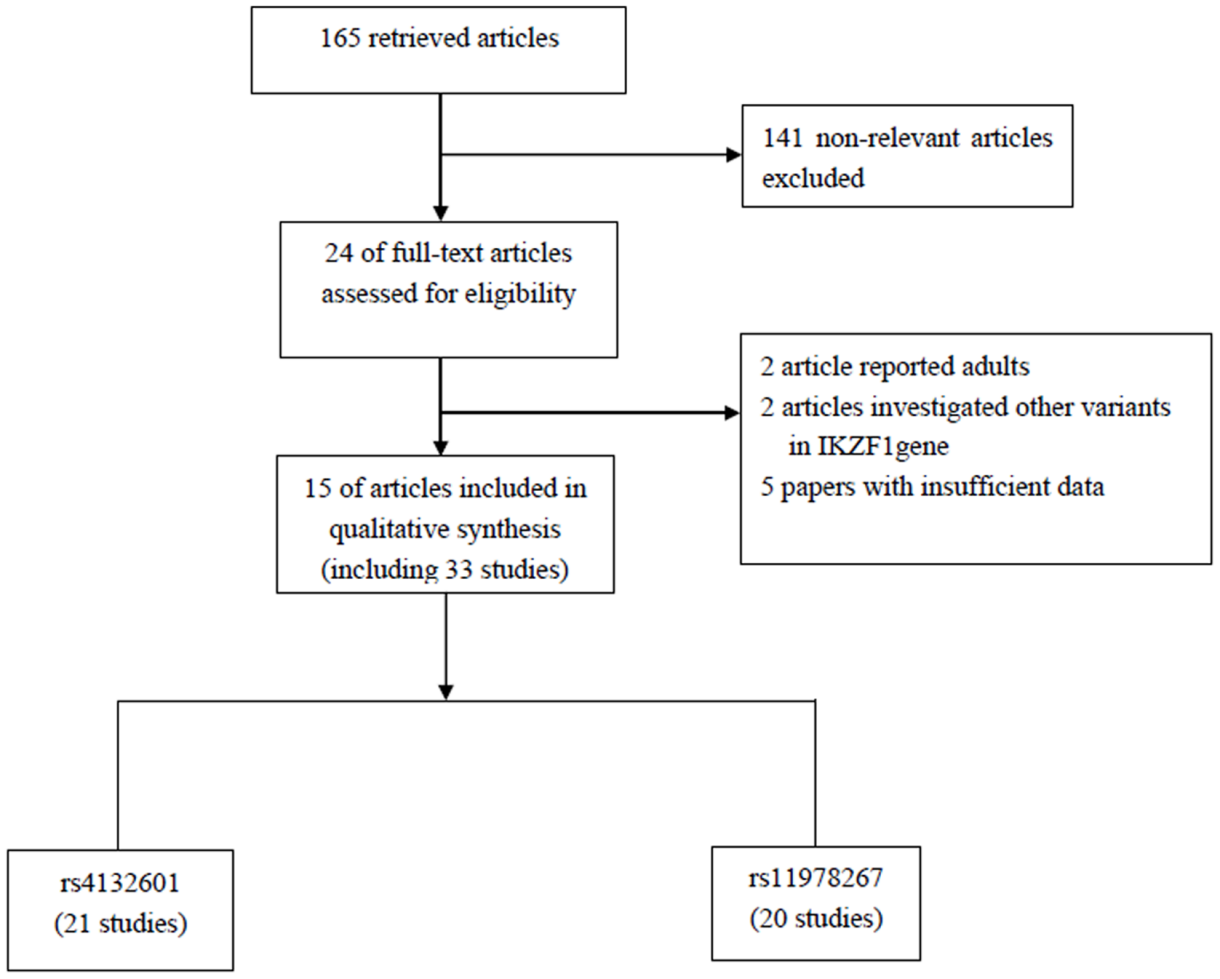
Flow diagram of study selection process.

The combined search yielded 165 references from PubMed, Embase, EBSCO, and Web of Science databases. After review of titles and abstracts, 141non-relevant articles were excluded, including review articles, meta-analysis, articles only with an abstract, and duplicate studies. Full texts of the remaining 24 articles were reviewed and analyzed in detail, of which, 2 articles reported in adults, 2 investigated other variants in IKZF1 gene, and 5 did not have sufficient data. Finally, a total of 15 relevant articles involving the associations between polymorphisms in IKZF1 and risk of childhood AL were eligible for this meta-analysis [5]–[13], [25]–[30]. Among them, 8 papers reported separate data of different diseases types (e.g., BCP-ALL, T-cell ALL or AML) and 4 articles reported separate data of different subpopulations, thus we treated them separately. Finally, a total of 33 studies comprising 9136 cases and 34748 controls were considered in our meta-analysis. The flow chart for the study selection process is shown in Figure 1, and the characteristics of all included studies are summarized in Table 1.

**Table 1:** Characteristics of studies included in the meta-analysis.

Variants	First author	Year	Country	Ethnicity	Disease	Genotyping Methods	Case/Control	Quality score
**rs4132601**	Papaemmanuil*	2009	UK	Europeans	B-cell ALL	Illumina Infinium Human 370Duo BeadChips	459/1438	11
**(T>G)**	Papaemmanuil#	2009	UK	Europeans	B-cell ALL	Illumina Infinium Human 370Duo BeadChips	365/960	11
	Lautner-Csorba	2012	Hungary	Europeans	B-cell ALL	Sequenom iPLEX Gold MassARRAY	390/529	10
	Lin	2014	China	Asians	B-cell ALL	TaqMan	45/80	6
	Prasad	2010	Germany	Europeans	B-cell ALL	Kaspar allele-specific PCR	1193/1516	11
	Prasad	2010	UK	Europeans	B-cell ALL	Kaspar allele-specific PCR	191/361	11
	Vijayakrishnan	2010	Thailand	Asians	B-cell ALL	Kaspar allele-specific PCR	172/182	9
	Ellinghaus	2012	Germany	Europeans	B-cell ALL	SNPlex and TaqMan	419/474	10
	Ellinghaus	2012	Germany	Europeans	B-cell ALL	SNPlex and TaqMan	406/1682	10
	Ellinghaus	2012	Italy	Europeans	B-cell ALL	SNPlex and TaqMan	287/579	10
	Healy	2010	Canada	Europeans	B-cell ALL	allele-specific primer extension	284/270	8
	Oris	2012	France	Europeans	B-cell ALL	Human CNV370-Quad Illumina beadchip	361/1542	9
	Papaemmanuil*	2009	UK	Europeans	T-cell ALL	Illumina Infinium Human 370Duo BeadChips	44/1438	11
	Papaemmanuil#	2009	UK	Europeans	T-cell ALL	Illumina Infinium Human 370Duo BeadChips	39/960	11
	Lautner-Csorba	2012	Hungary	Europeans	T-cell ALL	Sequenom iPLEX Gold MassARRAY	78/529	10
	Lin	2014	China	Asians	T-cell ALL	TaqMan	32/80	6
	Vijayakrishnan	2010	Thailand	Asians	T-cell ALL	Kaspar allele-specific PCR	18/182	9
	Oris	2012	France	Europeans	T-cell ALL	Human CNV370-Quad Illumina beadchip	41/1542	9
	Wang	2013	China	Asians	ALL	SNaPshot	570/673	11
	Pastorczak	2011	Poland	Europeans	ALL	TaqMan	398/731	10
	Rudant	2013	France	Europeans	AML	Illumina 370K Quad BeadChip	51/414	9
								
**rs11978267**	Xu	2013	USA	Europeans	B-cell ALL	Affymetrix GeneChip	574/2601	11
**(A>G)**	Xu	2013	USA	Africans	B-cell ALL	Affymetrix GeneChip	128/1075	11
	Xu	2013	USA	Europeans	B-cell ALL	Affymetrix GeneChip	143/640	11
	Emerenciano	2014	Brasil	Mixed	B-cell ALL	Taqman	77/490	9
	Emerenciano	2014	Brasil	Mixed	B-cell ALL	Taqman	77/490	9
	Treviño	2009	USA	Europeans	B-cell ALL	Affymetrix 500K Array Set chips	274/17958	10
	Healy	2010	Canada	Europeans	B-cell ALL	allele-specific primer extension	284/270	8
	Ellinghaus	2012	Germany	Europeans	B-cell ALL	SNPlex and TaqMan	419/474	10
	Ellinghaus	2012	Germany	Europeans	B-cell ALL	SNPlex and TaqMan	406/1682	10
	Ellinghaus	2012	Italy	Europeans	B-cell ALL	SNPlex and TaqMan	287/579	10
	Lautner-Csorba	2012	Hungary	Europeans	B-cell ALL	Sequenom iPLEX Gold MassARRAY	390/529	10
	Linabery	2013	USA	Europeans	B-cell ALL	Taqman	574/384	10
	Oris	2012	France	Europeans	B-cell ALL	Human CNV370-Quad Illumina beadchip	361/1542	9
	Oris	2012	France	Europeans	T-cell ALL	Human CNV370-Quad Illumina beadchip	41/1542	9
	Lautner-Csorba	2012	Hungary	Europeans	T-cell ALL	Sequenom iPLEX Gold MassARRAY	78/529	10
	Linabery	2013	USA	Europeans	T-cell ALL	Taqman	95/384	10
	Treviño	2009	USA	Europeans	T-cell ALL	Affymetrix 500K Array Set chips	44/17958	10
	Ross	2013	USA	Europeans	ALL	Taqman	96/384	9
	Ross	2013	USA	Europeans	AML	Taqman	62/384	9
	Emerenciano	2014	Brasil	Mixed	AML	Taqman	93/490	9

ALL: acute lymphoid leukemia; AML: acute myelogenous leukemia;

*: GWAS-1;

# : GWAS-2.

### Association of rs4132601 risk of childhood acute leukemia

**Figure 2:**
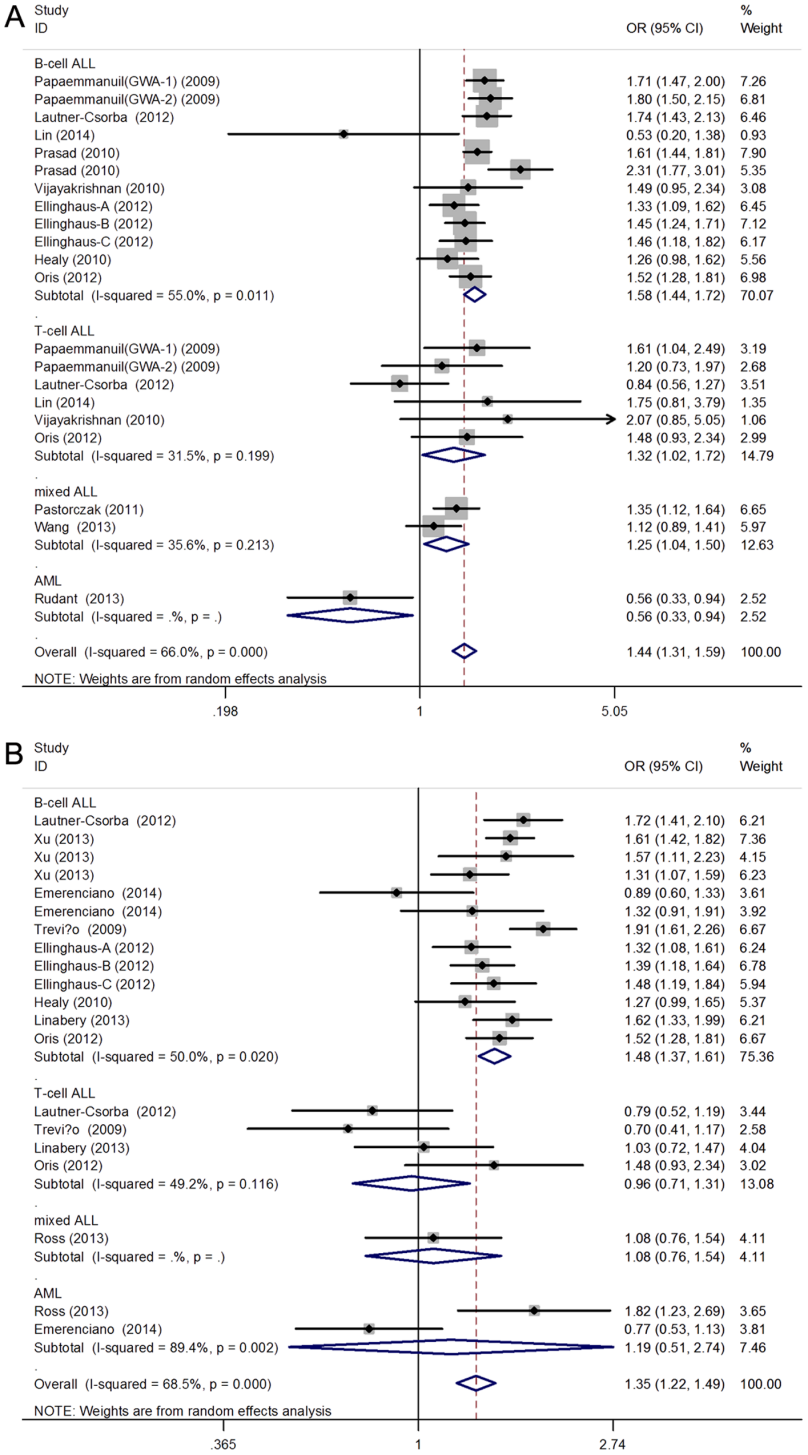
Forest plots of the association between IKZF1 gene polymorphisms: (A) rs4132601 polymorphism or (B) rs11978267 polymorphism and childhood AL risk in allelic contrast model (G vs T for rs4132601and G vs A for rs11978267). The sizes of the squares reflect the weighting of the included studies. Bars represent 95% CIs. The center of the diamond represents the summary effect; left and right pointsof the diamond represent the 95% CI. CI: confidence interval; OR: Odds ratio.

The association between rs4132601 polymorphism and susceptibility to AL was analyzed in21 studies involving 5823 AL patients and 11393 healthy controls. Overall, the results of combined analyses showed a significantly increased risk of AL in all genetic models. (G vs T: OR = 1.44, 95%CI = 1.31, 1.59, p<0.001; GG vs TT: OR = 2.23, 95%CI = 1.71, 2.90, P<0.001; GT vs TT: OR = 1.42, 95%CI = 1.21, 1.67; GG vs GT+TT: OR = 1.88, 95%CI = 1.52, 2.32, p<0.001; GG+GT vs TT: OR = 1.49, 95%CI = 1.25, 1.78, p<0.01) (Table 2 and Figure 2A) In the subgroup analysis stratified by types of AL, significant association was observed in BCP-ALL subgroup, but not among T-cell ALL, or AML subgroups. Moreover, in Europeans, persons with a G allele had a markedly increased risk of AL (G vs T: OR = 1.48, 95%CI = 1.34, 1.63, p<0.001), which was not observed in Asians (G vs T: OR = 1.44, 95%CI = 0.93, 1.73, p = 0.132). When stratified by source of control, significant association was observed in all genetic models in PB control subgroup.

**Table 2:** Pooled ORs and 95% CIs for associations between IKZF1 rs4132601 and rs11978267 polymorphisms and childhood AL risk.

Study group	G vs T			GG vs TT			GT vs TT			GG+GT vs TT			GG vs GT+TT		
*rs4132601*	OR(95%CI)	P_h_	P	OR(95%CI)	P_h_	P	OR(95%CI)	P_h_	P	OR(95%CI)	P_h_	P	OR(95%CI)	P_h_	P
**Total**	1.44(1.31,1.59)	<0.01	<0.01	2.23(1.71,2.90)	<0.01	<0.01	1.42(1.21,1.67)	<0.01	<0.01	1.49(1.25,1.78)	<0.01	<0.01	1.88(1.52,2.32)	0.03	<0.01
**Type**															
ALL	1.49(1.36,1.62)	<0.01	<0.01	2.37(1.87,3.00)	0.02	<0.01	1.49(1.28,1.73)	<0.01	<0.01	1.59(1.35,1.86)	<0.01	<0.01	1.94(1.60,2.36)	0.08	<0.01
BCP-ALL	1.57(1.44,1.72)	0.01	<0.01	2.67(2.17,3.29)	0.17	<0.01	1.67(1.40,1.99)	0.01	<0.01	1.82(1.52,2.17)	<0.01	<0.01	2.05(1.77,2.38)	0.46	<0.01
T-cell ALL	1.32(1.02,1.72)	0.20	0.03	1.84(0.48,7.02)	0.01	0.37	1.24(0.91,1.68)	0.80	0.18	1.27(0.95,1.70)	0.64	0.11	1.74(0.45,6.70)	<0.01	0.42
AML	0.56(0.33,0.94)	-	0.03	0.33(0.08,1.46)	-	0.14	0.56(0.29,1.06)	-	0.07	0.52(0.28,0.95)	-	0.03	0.42(0.10,1.78)	-	0.24
**Ethnicity**															
Europeans	1.48(1.34,1.63)	<0.01	<0.01	2.23(1.70,2.92)	<0.01	<0.01	1.50(1.26,1.78)	<0.01	<0.01	1.58(1.31,1.91)	<0.01	<0.01	1.87(1.51,2.31)	0.03	<0.01
Asians	1.27(0.93,1.73)	0.17	0.13	2.93(0.59,14.5)	0.13	0.19	1.19(0.83,1.70)	0.17	0.34	1.24(0.89,1.75)	0.18	0.20	2.88(0.56,14.85)	0.12	0.21
**Control**															
PB	1.50(1.35,1.67)	<0.01	<0.01	2.36(1.76,3.15)	<0.01	<0.01	1.54(1.30,1.81)	<0.01	<0.01	1.62(1.35,1.94)	<0.01	<0.01	1.93(1.52,2.46)	0.02	<0.01
HB	1.26(1.04,1.52)	0.15	0.02	1.60(1.01,2.53)	0.98	0.04	1.11(0.83,1.48)	0.22	0.47	1.16(0.87,1.55)	0.20	0.32	1.51(0.97,2.33)	0.89	0.07
***rs11978267***	**G vs A**			**GG vs AA**			**GA vs AA**			**GG+GA vs AA**			**GG vs GA+AA**		
**Total**	1.35(1.22,1.49)	<0.01	<0.01	1.81(1.39,2.37)	0.01	<0.01	1.26(1.08,1.46)	0.01	<0.01	1.32(1.12,1.56)	<0.01	<0.01	1.67(1.33,2.10)	0.04	<0.01
**Type**															
ALL	1.37(1.24,1.51)	<0.01	<0.01	1.85(1.42,2.39)	0.03	<0.01	1.35(1.15,1.60)	0.05	<0.01	1.38(1.17,1.62)	<0.01	<0.01	1.67(1.35,2.06)	0.14	<0.01
BCP-ALL	1.48(1.37,1.61)	0.02	<0.01	2.08(1.65,2.63)	0.14	<0.01	1.48(1.32,1.66)	0.33	<0.01	1.56(1.35,1.79)	0.11	<0.01	1.78(1.48,2.15)	0.31	<0.01
T-cell ALL	0.96(0.71,1.31)	0.12	0.81	0.53(0.07,3.96)	0.06	0.54	1.02(0.73,1.44)	0.74	0.90	0.96(0.69,1.34)	0.81	0.81	0.52(0.06,4.18)	0.05	0.54
AML	1.18(0.51,2.74)	<0.01	0.69	1.74(0.33,9.04)	<0.01	0.51	0.86(0.55,1.37)	0.23	0.53	1.04(0.48,2.26)	0.03	0.91	1.81(0.42,7.89)	0.01	0.43
**Ethnicity**															
Europeans	1.41(1.27,1.55)	<0.01	<0.01	2.07(1.58,2.73)	0.03	<0.01	1.31(1.12,1.54)	0.04	<0.01	1.40(1.18,1.67)	0.01	<0.01	1.84(1.44,2.35)	0.06	<0.01
African	1.57(1.11,2.23)	-	0.01	2.07(0.79,5.46)	-	0.14	1.72(1.10,2.70)	-	0.02	1.76(1.14,2.71)	-	0.01	1.68(0.65,4.35)	-	0.28
mixed	0.97(0.71,1.34)	0.12	0.87	0.99(0.56,1.75)	0.32	0.97	0.98(0.67,1.42)	0.19	0.90	0.97(0.66,1.44)	0.13	0.89	0.99(0.59,1.67)	0.49	0.98
**Control**															
PB	1.42(1.27,1.57)	<0.01	<0.01	2.16(1.64,2.84)	0.05	<0.01	1.35(1.14,1.59)	0.04	<0.01	1.44(1.21,1.72)	0.01	<0.01	1.90(1.48,2.43)	0.08	<0.01
HB	1.15(0.91,1.45)	0.02	0.24	1.21(0.78,1.87)	0.30	0.39	1.06(0.80,1.40)	0.22	0.69	1.07(0.78,1.45)	0.11	0.68	1.19(0.82,1.72)	0.50	0.35

AL: acute leukemia; ALL: acute lymphoid leukemia; AML: acute myelogenous leukemia; CI: confidence interval; OR: Odds ratio; P_h_: P value for heterogeneity.

### Association of rs11978267 with risk of childhood acute leukemia

A total of 20 studies with 4960 patients and 28034 controls were eligible for the pooled analysis of rs11978267 polymorphism. Meta-analysis findings of associations between rs11978267 polymorphism in IKZF1 gene and susceptibility of acute leukemia were summarized in Table 2. Significantly increased AL risk was observed in all comparisons. (G vs A: OR = 1.35, 95%CI = 1.22, 1.49, p<0.001) (Figure 2B) When stratified by types of AL, significant correlation was found in BCP-ALL subgroup in the allelic and all genetic models. However, these associations were not statisticaly significant in T-cell ALL and AML subgroups. When performing meta-analysis by ethnicity, higher risk can be detect in Europeans, but not in African and mixed populations. In the subgroup analysis stratified analysis, a significantly increased childhood AL risk was found in PB control subgroup (G vs A: OR = 1.42, 95%CI = 1.27, 1.57, p<0.01) but not among HB control subgroup. (G vs A: OR = 1.15, 95%CI = 0.91, 1.45, p = 0.24)

### Test of heterogeneity

Heterogeneity was significant in most comparisons of the two IKZF1 SNPs. The results of meta-regression suggested that types of disease might be a potential source of heterogeneity, which could explain 86.7% and 65.9% of τ^2^ in the analysis of rs11978267 and rs4132601 polymorphism, respectively. In addition, the heterogeneity was removed in T-cell ALL or Asians subgroup of rs4132601 variant and in BCP-ALL, T-cell ALL or mixed population subgroup of rs11978267 variant. (Table 2) We further performed Galbraith plot analyses, which indicated that 5 and 4 studies were the possible origin of heterogeneity for rs11978267 and rs4132601 variants, respectively, when excluded, the heterogeneity was removed and the association was still significant. (Figure S1)

### Sensitivity analysis and cumulative meta-analysis

**Figure 3:**
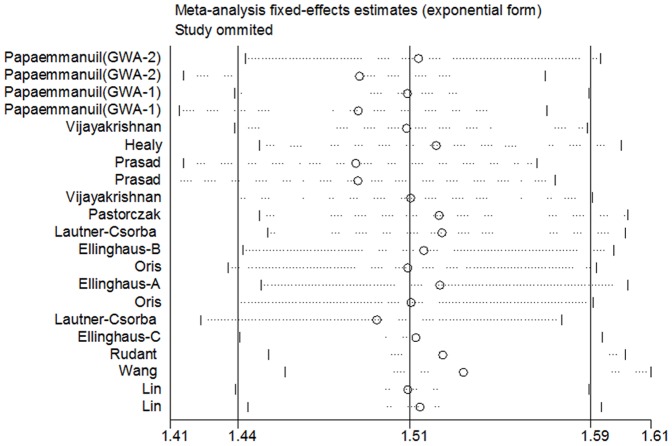
Sensitivity analysis on the associations between IKZF1 rs4132601 variant and childhood AL risk in allelic contrast model (G vs T). Results were computed by omitting each study (left column) in turn, Bars: 95% confidence interval.

**Figure 4:**
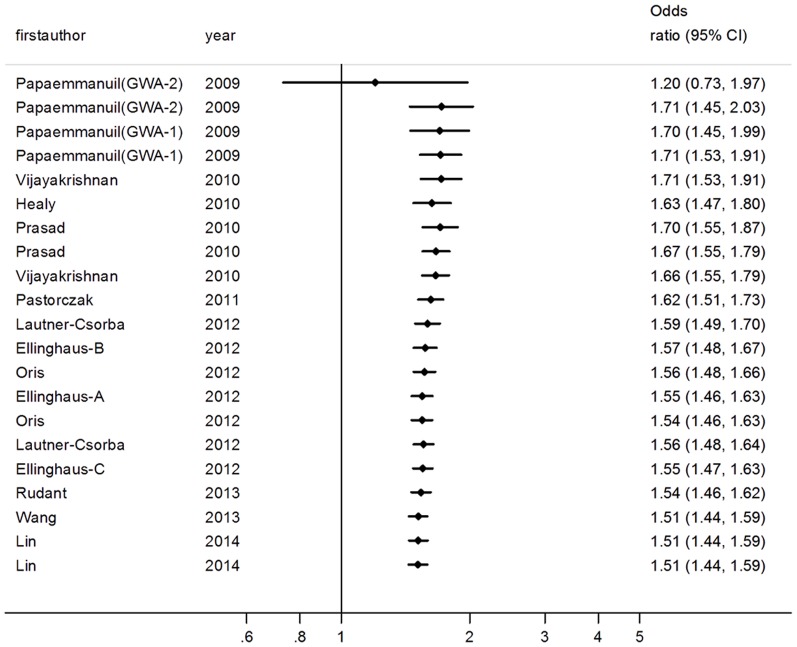
Cumulative meta-analysis: pooled OR with the corresponding 95% CI at the end of each year information step is shown for IKZF1 rs4132601 polymorphism in allelic contrast model (G vs T). CI: confidence interval; OR: Odds ratio.

For rs4132601 polymorphism, sensitivity analysis indicated that no single study qualitatively changed the pooled ORs. (Figure 3) In the cumulative meta-analysis, the pooled ORs tended to be stable as more data accumulating over time. (Figure 4) Similar results of sensitivity analysis and cumulative meta-analysis were observed in the analysis of rs11978267 polymorphism. (Figure S2 and Figure S3) Together, these results suggested results of this meta-analysis were highly stable.

### Test of publication bias

**Figure 5:**
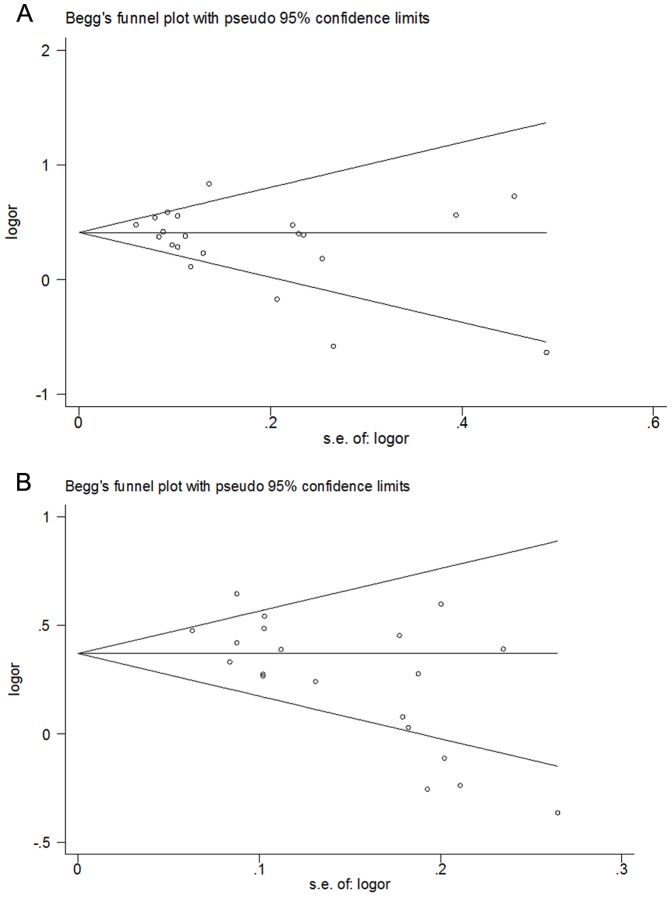
Publication bias in studies of the association between IKZF1 rs4132601 polymorphism and childhood AL risk assessed by Begg's Funnel plot (G vs T). No significant funnel asymmetry was observed which could indicate publication bias. The horizontal line in the funnel plot indicates the random effects summary estimate, while the sloping lines indicate the expected 95% CI for a given standard error, assuming no heterogeneity between studies. Logor: natural logarithm of the OR.

Funnel plots and Egger's test were carried out to assess publication bias. The shapes of the funnel plots did not indicate any evidence of obvious asymmetry for rs4132601 variant, which was supported by the Egger's test. (GG vs TT: p = 0.25) For rs11978267 variant, however, significant publication bias was detected in most comparisons (GG vs TT: p = 0.01). (Figure 5 and Table S2)

## Discussion

IKZF1, located on chromosome 7p12.2,is an essential regulator of lymphopoiesis and plays an important role in the development of lymphoid lineages, especially in the commitment of CD4 and CD8 T-cell lineages [31], [32]. Previous studies have demonstrated that loss of IKZF1 resulted in haploinsufficiency, expression of a dominant-negative Ikaros isoform, or the complete loss of Ikaros expression [33]. Moreover, IKZF1 deletions were associated with poor prognosis in childhood BCP-ALL [34], [35]. Thus, variants in the IKZF1 gene maybe associated with increased risk of childhood acute leukemia. Although a number of studies reported associations between IKZF1 polymorphisms and AL risk, the results were inconsistent.

In this meta-analysis, we observed a significantly increased AL risk in the analysis of rs4132601 polymorphism in IKZF1 gene in all genetic contrasts. When the data were stratified by disease type, a significant association was found in BCP-ALL subgroup, but not among T-cell ALL or AML subgroups. Similar results were found with thers11978267 variant. It is widely accepted that childhood ALisnot a single homogeneous disease and canbeclassified into subtypes: acute lymphoblastic (ALL) and myeloid leukaemia (AML), eachwith their own characteristics and potentially different aetiologies [36], [37]. Also, the incidence of childhood ALL is approximately five times more frequent than AML [38]. Moreover, previous studies have demonstrated that genetic polymorphisms might have a different effect on the susceptibility of various subtypes of AL. This observation was also supported by the findings that XRCC1 Arg399Glnvariantwas associated with risk of ALL, but not with AML risk (ref).

In the subgroup analysis by ethnicity, statistical correlation was observed in Europeans for both variants. However, no significantly increased ALL risk was found in Asians forrs4132601 polymorphism and African or mixed populations for rs11978267 polymorphism, suggesting that the relative contribution of susceptibility genes may vary across different ethnicities. International variation in the incidence of leukaemia, especially ALL, is well recognized, which was 44% higher among Whites compared to Blacks (27/106 person-years vs 15/106 person-years, P<0.0001) [39]. Variations in environmental exposures and genetic susceptibility can account for, at least partially, differences in childhood leukemia incidence rates. In addition, the difference might also be attributed to that early infectious insulation, in developed countries, predisposes the immune system of individuals to aberrant responses after subsequently delayed antigenic stimulation, which has been proposed as a cause of common ALL [40].

Significant between-study heterogeneity existed when all 27 studies were pooled. We found the heterogeneity was remarkably decreased or even removed among Asian or mixed population subgroups, T-cell ALL subgroup and BCP-ALL subgroup. We then performed meta-regression to explore the potential source contributing to the heterogeneity, which suggested that types of disease might be a potential source of heterogeneity. The results indicated that disease type could explain 86.7% of τ^2^ in the analysis of rs11978267 polymorphism. Moreover, the heterogeneity was removed in BCP-ALL, T-cell ALL and AML subgroups under heterozygote comparison. (GA vs AA: Ph = 0.33, Ph = 0.74, and Ph = 0.23, respectively) Furthermore, Galbraith plot analyses was also carried out to visualize the impact of individual studies on the overall heterogeneity, which indicated that 5 studies were the possible origin of heterogeneity, when excluded, the heterogeneity was removed and the association was still significant. (G vs A: OR = 1.47, 95%CI = 1.39, 1.55, p<0.01, Ph = 0.21) In addition, sensitivity analysis showed that no single study qualitatively changed the pooled odds ratios. Also, results of cumulative meta-analysis showed the pooled ORs tended to be stable and the associations tended toward significant as more data accumulating over time, indicating that the results of this meta-analysis are stable.

Several limitations of our study should be acknowledged. First, only1 and 5 studies were included in Africans and Asians, respectively. Thus, the association between IKZF1 polymorphisms and childhood acute leukemia in different populations need to be validated by further studies with large sample size. Second, a language bias may have occurred because of only studies published in English were included. Also, significant heterogeneity between studies was detected in this meta-analysis. Finally, the etiology of childhood ALL is believed to be multi-factorial, including genetic variables, infections, and environmental risk factors such as ionizing radiation. However, we could not perform gene-environment or gene-gene interactions due to the insufficient data.

In summary, despite these limitations, our results were still significant. The results of this meta-analysis suggested that rs4132601 and rs11978267 polymorphisms in IKZF1 gene might contribute to the occurrence of BCP-ALL, especially in European populations. However, the association in other ethnic groups (e.g., Asians and Africans) needs to be validated in further studies with large sample size. Moreover, studies involving gene-gene and gene-environment interactions are required to clarify possible roles of multiple risk factors in childhood AL.

## Supporting Information

Figure S1 **Sensitivity analysis on the associations between IKZF1 rs11978267 variant and childhood AL risk in allelic contrast model (G vs A).** Results were computed by omitting each study (left column) in turn, Bars: 95% confidence interval.
(TIF)

Figure S2 **Cumulative meta-analysis: pooled OR with the corresponding 95% CI at the end of each year information step is shown for IKZF1 rs11978267 polymorphism in allelic contrast model (G vs A).** CI: confidence interval; OR: Odds ratio.
(TIF)

Figure S3 **Galbraith plots of IKZF1 rs4132601 (A) or rs11978267 (B) polymorphism and childhood AL risk, which indicated the outliers as possible sources of heterogeneity.** The regression runs through the origin interval (central solid line). The 95% confidence interval is between the two outer parallel lines at two units above and below the regression line.
(TIF)

Table S1 **Scale for quality assessment.**
(DOC)

Table S2 **P value for the Egger's test in the analysis of publication bias.**
(DOC)

Checklist S1 **PRISMA checklist.**
(DOC)
