# Supplementary figures and images for: Contribution of Polymorphisms in IKZF1 Gene to Childhood Acute Leukemia: A Meta-Analysis of 33 Case-Control Studies
Source: PLoS One. 2014 Nov 25;9(11):e113748. doi: 10.1371/journal.pone.0113748 (PMC4244140; doi:10.1371/journal.pone.0113748)

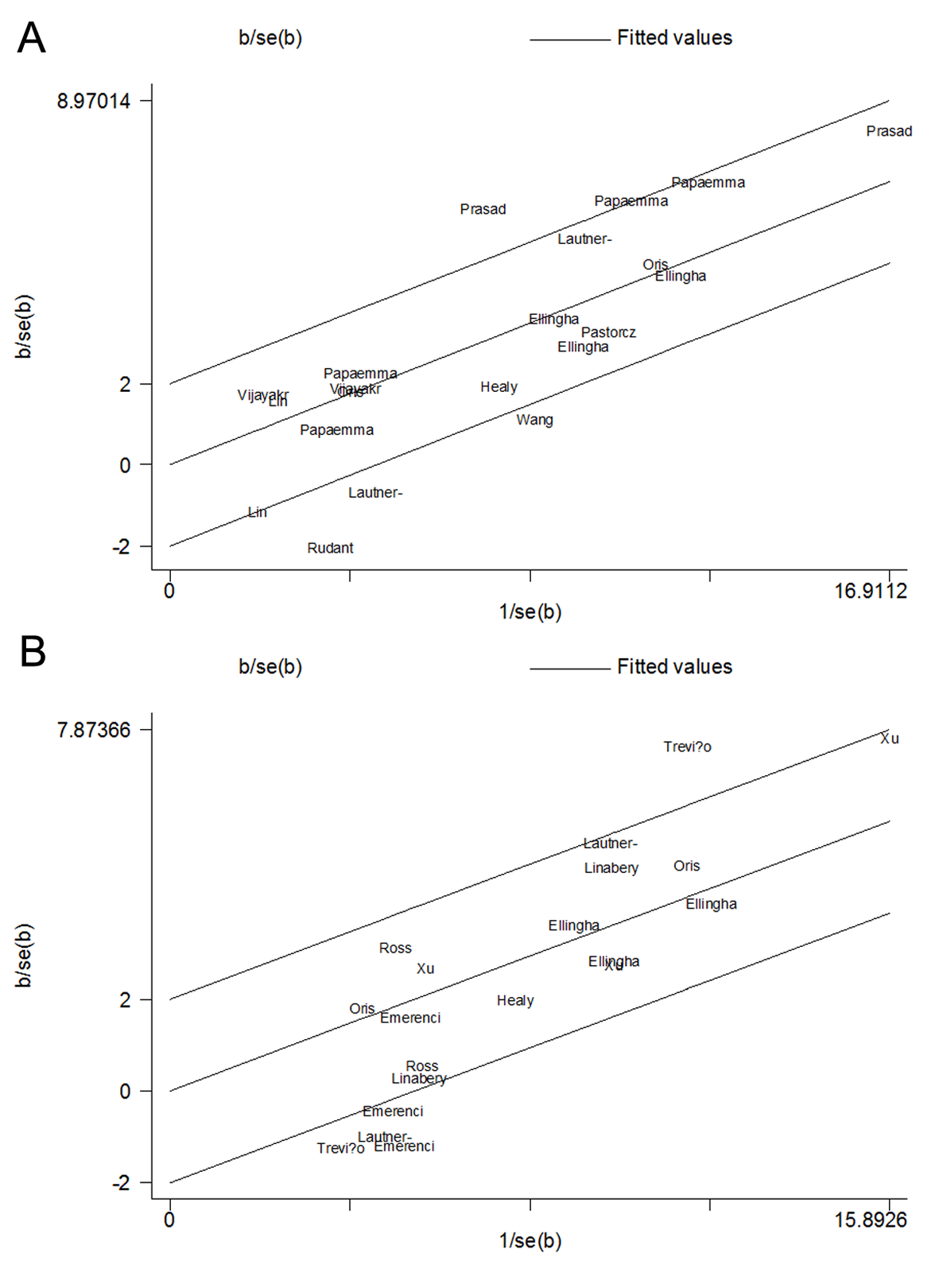

Supplement: Figure S1 — Sensitivity analysis on the associations between IKZF1 rs11978267 variant and childhood AL risk in allelic contrast model (G vs A). Results were computed by omitting each study (left column) in turn, Bars: 95% confidence interval. (TIF) [file pone.0113748.s001.tif]

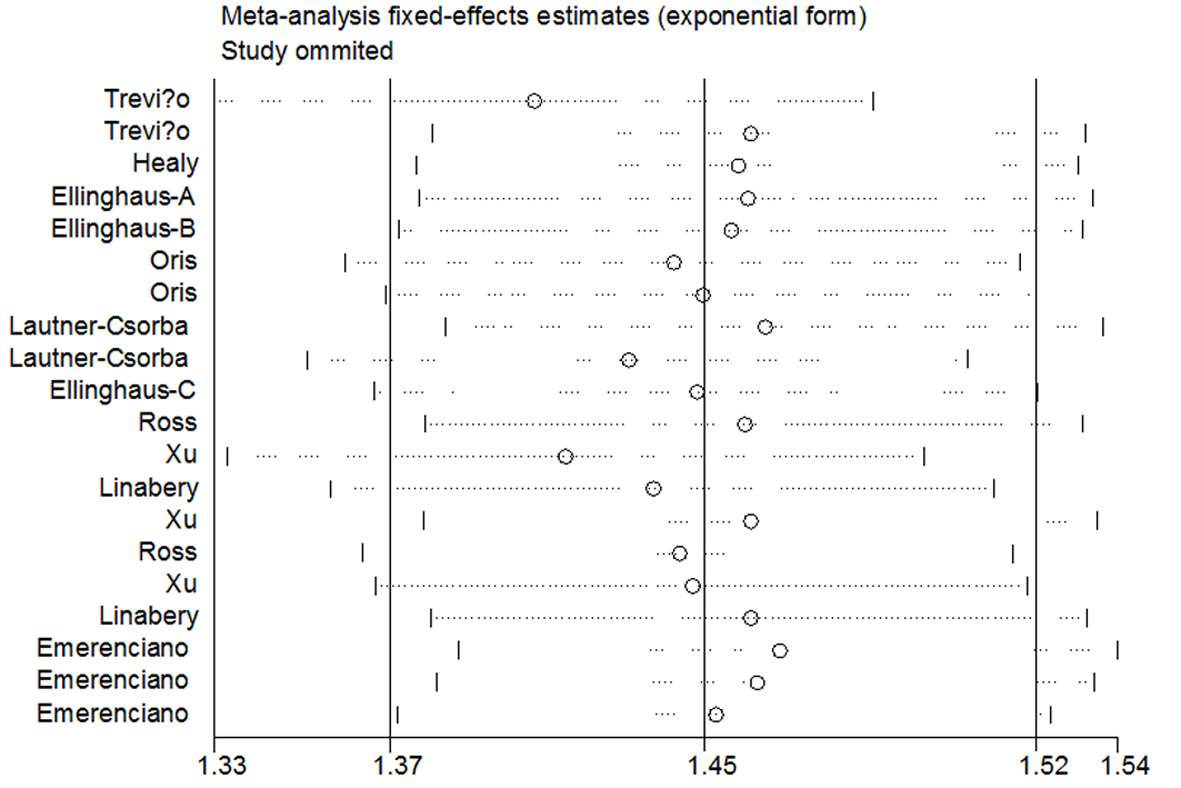

Supplement: Figure S2 — Cumulative meta-analysis: pooled OR with the corresponding 95% CI at the end of each year information step is shown for IKZF1 rs11978267 polymorphism in allelic contrast model (G vs A). CI: confidence interval; OR: Odds ratio. (TIF) [file pone.0113748.s002.tif]

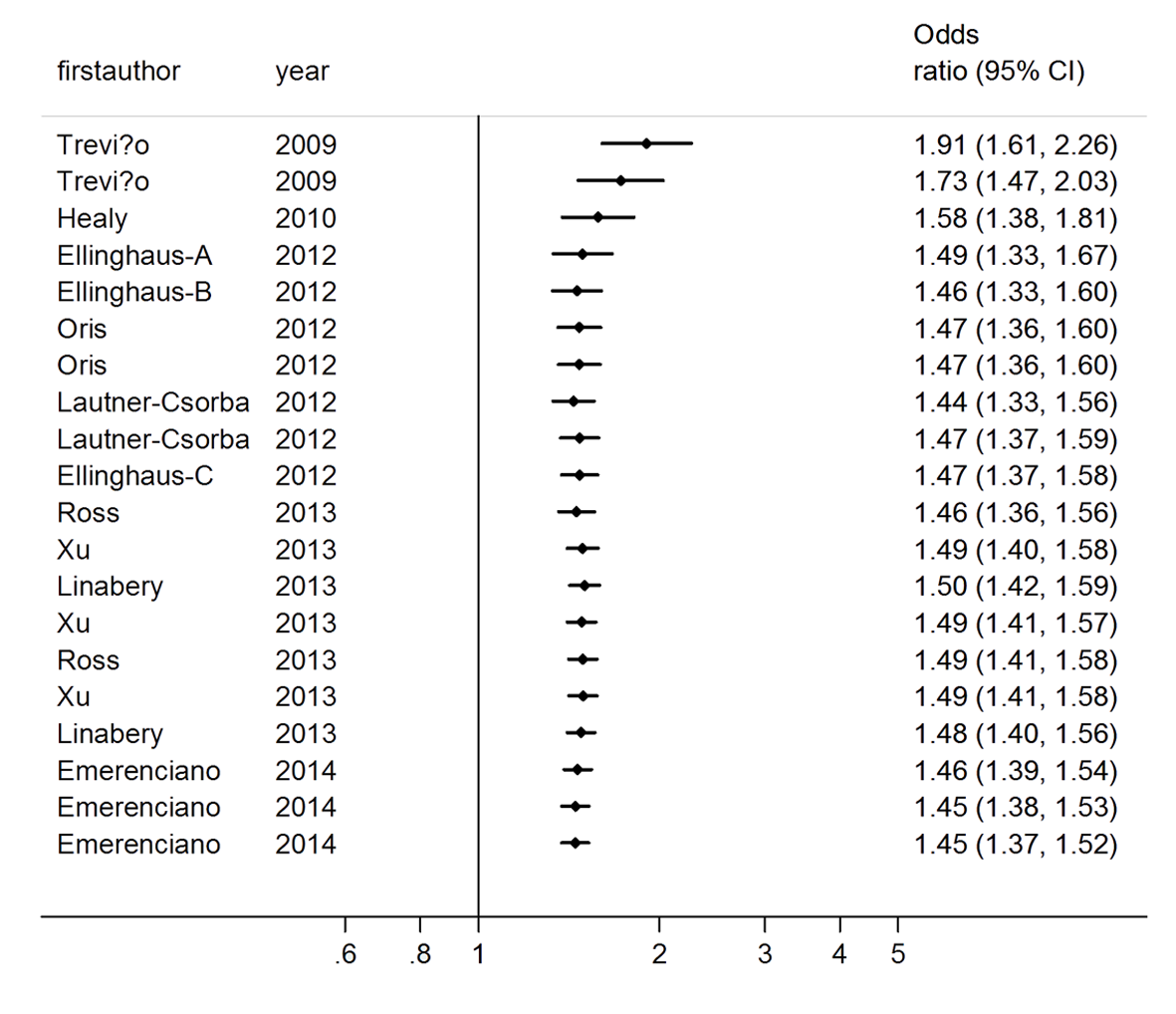

Supplement: Figure S3 — Galbraith plots of IKZF1 rs4132601 (A) or rs11978267 (B) polymorphism and childhood AL risk, which indicated the outliers as possible sources of heterogeneity. The regression runs through the origin interval (central solid line). The 95% confidence interval is between the two outer parallel lines at two units above and below the regression line. (TIF) [file pone.0113748.s003.tif]
