# Supplementary material for: Contribution of Polymorphisms in IKZF1 Gene to Childhood Acute Leukemia: A Meta-Analysis of 33 Case-Control Studies
Source: PLoS One. 2014 Nov 25;9(11):e113748. doi: 10.1371/journal.pone.0113748 (PMC4244140; doi:10.1371/journal.pone.0113748)
Supplement: Table S1 — Scale for quality assessment. (DOC) [file pone.0113748.s004.doc]

**Table S1: Scale for quality assessment**

| Criteria | Score |
| --- | --- |
| Representativeness of cases |  |
| Consecutive/randomly selected from case population with clearly defined sampling frame | 2 |
| Consecutive/randomly selected from case population without clearly defined sampling frame | 1 |
| Not described | 0 |
| Source of controls |  |
| Population- or neighbor-based | 2 |
| Hospital-based | 1 |
| Not described | 0 |
| Hardy-Weinberg equilibrium in controls |  |
| Hardy-Weinberg equilibrium | 2 |
| Hardy-Weinberg disequilibrium | 1 |
| Genotyping examination |  |
| Genotyping done under “blinded” condition | 1 |
| Unblinded or not mentioned | 0 |
| Association assessment |  |
| Assess association between genotypes and child AL with appropriate statistics and adjustment for confounders | 2 |
| Assess association between genotypes and child AL with appropriate statistics without adjustment for confounders | 1 |
| Inappropriate statistics used | 0 |
| Total sample size |  |
| ≥1000 | 3 |
| ≥500 but <1000 | 2 |
| ≥200 but <500 | 1 |
| <200 | 0 |
