# Supplementary material for: Contribution of Polymorphisms in IKZF1 Gene to Childhood Acute Leukemia: A Meta-Analysis of 33 Case-Control Studies
Source: PLoS One. 2014 Nov 25;9(11):e113748. doi: 10.1371/journal.pone.0113748 (PMC4244140; doi:10.1371/journal.pone.0113748)
Supplement: Table S2 — P value for the Egger's test in the analysis of publication bias. (DOC) [file pone.0113748.s005.doc]

**Table S2:** P value for the Egger’s test in the analysis of publication bias.

| ***rs4132601*** | **G vs T** | **GG vs TT** | **GT vs TT** | **GG+GT vs TT** | **GG vs GT+TT** |
| --- | --- | --- | --- | --- | --- |
| **Total** | 0.09 | 0.25 | 0.12 | 0.12 | 0.37 |
| **Type** |  |  |  |  |  |
| ALL | 0.21 | 0.51 | 0.25 | 0.26 | 0.68 |
| B-cell ALL | 0.31 | 0.99 | 0.44 | 0.41 | 0.91 |
| T-cell ALL | 0.21 | 0.97 | 0.73 | 0.12 | 0.99 |
| AML | - | - | - | - | - |
| **Ethnicity** |  |  |  |  |  |
| Europeans | 0.07 | 0.09 | 0.14 | 0.13 | 0.09 |
| Asians | 0.70 | 0.62 | 0.99 | 0.83 | 0.64 |
| **Control** |  |  |  |  |  |
| PB | 0.20 | 0.36 | 0.15 | 0.18 | 0.48 |
| HB | 0.59 | - | 0.92 | 0.88 | - |
|  |  |  |  |  |  |
| ***rs11978267*** | **G vs A** | **GG vs AA** | **GA vs AA** | **GG+GA vs AA** | **GG vs GA+AA** |
| **Total** | 0.002 | 0.01 | 0.003 | 0.004 | 0.06 |
| **Type** |  |  |  |  |  |
| ALL | 0.001 | 0.004 | 0.01 | 0.004 | 0.02 |
| B-cell ALL | 0.08 | 0.09 | 0.11 | 0.07 | 0.17 |
| T-cell ALL | 0.81 | - | - | - | - |
| AML | - | - | - | - | - |
| **Ethnicity** |  |  |  |  |  |
| Europeans | 0.01 | 0.09 | 0.003 | 0.01 | 0.31 |
| mixed | 0.60 | 0.42 | 0.24 | 0.48 | 0.26 |
| **Control** |  |  |  |  |  |
| PB | 0.03 | 0.12 | 0.02 | 0.04 | 0.34 |
| HB | 0.09 | 0.21 | 0.60 | 0.53 | 0.16 |
